# Multi-morbidities of allergic rhinitis in adults: European Academy of Allergy and Clinical Immunology Task Force Report

**DOI:** 10.1186/s13601-017-0153-z

**Published:** 2017-06-01

**Authors:** C. Cingi, P. Gevaert, R. Mösges, C. Rondon, V. Hox, M. Rudenko, N. B. Muluk, G. Scadding, F. Manole, C. Hupin, W. J. Fokkens, C. Akdis, C. Bachert, P. Demoly, J. Mullol, A. Muraro, N. Papadopoulos, R. Pawankar, P. Rombaux, E. Toskala, L. Kalogjera, E. Prokopakis, P. W. Hellings, J. Bousquet

**Affiliations:** 1 Department of Otorhinolaryngology, Eskisehir Osmangazi University School of Medicine, Eskisehir, Turkey; 2 Upper Airway Research Laboratory, Ghent University Hospital, Ghent, Belgium; 3 Institute of Medical Statistics, Informatics, and Epidemiology, Medical Faculty, University of Köln, Cologne, Germany; 4 Allergy Unit, IBIMA, Regional University Hospital of Malaga, UMA, Malaga, Spain; 5 Clinical division of Otorhinolaryngology, Head and Neck Surgery, University Hospitals Leuven, Louvain, Belgium; 6 London Allergy and Immunology Centre, London, UK; 7 ENT Department, Faculty of Medicine, Kirikkale University, Kirikkale, Turkey; 8 Royal National Throat, Nose and Ear Hospital, London, UK; 9 Faculty of Medicine, ENT Department, University of Oradea, Oradea, Romania; 10 Institut de Recherche Expérimentale et Clinique (IREC), Pole de Pneumologie, ORL & Dermatologie, Université catholique de Louvain, Louvain-la-Neuve, Belgium; 11 Department of Otorhinolaryngology, Head and Neck Surgery, Academic Medical Centre (AMC), Amsterdam, The Netherlands; 12 Christine Kuhne-Center for Allergy Research and Education, Swiss Institute of Allergy and Asthma Research, University of Zurich, Davos, Switzerland; 13 Hôpital Arnaud de Villeneuve, University Hospital of Montpellier, Montpellier, France; 14 Unitat de Rinologia i Clinica de l’Olfacte, Servei d’Otorinolaringologia, Hospital Clínic, Barcelona, Catalonia Spain; 15 The Referral Centre for Food Allergy Diagnosis and Treatment Veneto Region, Department of Mother and Child Health, University of Padua, Padua, Italy; 16 Allergy Department, 2nd Pediatric Clinic, University of Athens, Athens, Greece; 17 Nippon Medical School, Tokyo, Japan; 18 Service d’ORL, Cliniques Universitaires St-Luc, Brussels, Belgium; 19 Department of Otorhinolaryngology-Head and Neck Surgery, Temple University, Philadelphia, PA USA; 20 Department of Otorhinolaryngology and Head and Neck Surgery, University Hospital Sestre milosrdnice, Zagreb, Croatia; 21 Department of Otorhinolaryngology, University Hospital of Crete, Crete, Greece

**Keywords:** Adenoid hypertrophy, Allergic rhinitis (AR), Asthma, Chronic middle ear effusions, Comorbidities, Disordered sleep, Eczema, Eosinophilic oesophagitis (EoE), Conjunctivitis, Food allergies, Obstructive sleep apnea, Olfaction disorders, Rhinitis, Rhinosinusitis

## Abstract

This report has been prepared by the European Academy of Allergy and Clinical Immunology Task Force on Allergic Rhinitis (AR) comorbidities. The aim of this multidisciplinary European consensus document is to highlight the role of multimorbidities in the definition, classification, mechanisms, recommendations for diagnosis and treatment of AR, and to define the needs in this neglected area by a literature review. AR is a systemic allergic disease and is generally associated with numerous multi-morbid disorders, including asthma, eczema, food allergies, eosinophilic oesophagitis (EoE), conjunctivitis, chronic middle ear effusions, rhinosinusitis, adenoid hypertrophy, olfaction disorders, obstructive sleep apnea, disordered sleep and consequent behavioural and educational effects. This report provides up-to-date usable information to: (1) improve the knowledge and skills of allergists, so as to ultimately improve the overall quality of patient care; (2) to increase interest in this area; and (3) to present a unique contribution to the field of upper inflammatory disease.

## Introduction

This report was prepared by the European Academy of Allergy and Clinical Immunology (EAACI) Task Force on “Allergic Rhinitis (AR) comorbidities”. This was initiated, based on the rationale that AR is rarely found in isolation and needs to be considered in the context of systemic allergic disease associated with numerous comorbid disorders including asthma, chronic middle ear effusions, sinusitis, lymphoid hypertrophy with obstructive sleep apnea, disordered sleep, and consequent behavioural and educational effects.

AR, which has increased in prevalence over several decades, now affects 10–30% of the population, with the greatest frequency found in children and adolescents [1]. It typically presents after the second year of life but the exact prevalence in early life is unknown. Since children’s immune systems develop between the first and fourth years of life, those with an atopic predisposition begin to express allergic disease with a clear Th_2_ response to allergen exposure, resulting in symptoms, often beginning with atopic dermatitis (AD) and progressing to asthma and rhinitis (the allergic march) [1]. However after early childhood, AR is usually the initial manifestation of allergy [2].

AR is associated with numerous multi-morbid disorders. Those occurring in children have already been discussed in the EAACI Task Force on Paediatric Rhinitis [3]. This paper, by contrast, concerns itself largely with adult AR multimorbidities, but includes relevant paediatric data.

## Definition

Multimorbidity is the presence of one or more additional disorders (or diseases) *co*-*occurring* with a primary disease or disorder; or the effect of such additional disorders or diseases [4]. When the primary organ is not known, the term multimorbidity should be used instead of co-morbidity. In allergic diseases, the term should be multimorbidity.

## Multi-morbidities of allergic rhinitis

AR is an organ-specific manifestation of allergic disease. As such, it coexists with other organ-specific disorders that have a common allergic basis. It is therefore rarely found in isolation but frequently has associated multi-morbid disorders [5].

These can be subdivided into:

- Disorders which are part of the spectrum of allergic diseases, e.g. asthma, AD, food allergy, anaphylaxis;
- Disorders anatomically related to the nose: conjunctivitis, sinusitis, middle ear problems, throat and laryngeal effects;
- Sleep problems and secondary effects on concentration and behaviour; and
- Turbinate hypertrophy.

Although more common in paediatric practice, the occurrence of multi-morbidities in adults is significant and has important implications for quality of life, and work attendance and performance. It is likely that those with severe chronic upper airways disease (SCUAD) suffer more severe co-morbid effects.

### Asthma

#### Extent of co-occurrence

A European study of over 20,000 children showed that the co-existence of eczema, asthma and rhinitis in the same child is more frequent than expected if they were independent entities. Those children with one of these diseases at age 4 were 4–7 times more likely to have two or three of them at age 8. Children with two or three allergic diseases at 4 years are 30–60 times more likely to have two or three of these diseases at age 8 [6].

The association between asthma and rhinitis in adults has been recognized for some decades, since the pioneering study of Brydon [7], who showed that the majority of 1000 asthmatics also had rhinitis, which preceded their asthma in 45% of the subjects.

In fact the majority of inflammatory asthma sufferers have some form of upper airway disease (ARIA 2001) [8], either AR, non-allergic rhinitis or rhinosinusitis, usually with nasal polyposis (EPOS 2012) [9]. The extent of inflammation is usually proportional between upper and lower respiratory tracts with equivalence in eosinophils [10] and clinical severity [11].

Several possible relations exist between AR and asthma: (a) AR may be statistically associated with asthma; (b) AR may exacerbate coexisting asthma; and (c) AR may have a causal role in the pathogenesis of asthma.

Several possible causal mechanisms have been postulated to explain a link between AR and asthma:

- Lack of nasal function, i.e. purifying, warming and humidifying inspired air;
- Nasobronchial reflex (nasal irritants, allergens or cold stimuli);
- Rhinovirus adhesion theory (increased susceptibility to allergic inflammation and intracellular adhesion molecule (ICAM)-1 expression) [12]; and
- “Migration” of T cell responses to other tissues after initial sensitization. Braunstahl [13] has shown that allergen challenge in one part of the airway is followed by a response in all other parts;
- The idea of postnasal drip (carriage of inflammatory cytokines/mediators from nasopharynx to lower airways) has been largely abandoned, since the ‘drip’ travels to the gut, by virtue of the larynx, not to the lower airway, unless the subject is deeply unconscious.

Certainly the presence of rhinitis, both allergic and non-allergic, is a risk factor for subsequent asthma development [14].

#### Effects of co-occurrence

The co-existence of rhinitis is associated with poor asthma control in adults, adolescents and children [15]. Recent studies on AR and asthma are presented in Table 1 [16–19].

**Table 1:** Asthma and AR

References	Study type	No. patients	Age/Profile	Aim of the study	Results
Ciprandi et al. [16]	Prospective	89 (AR), 940 (controls)	Adults	Follow up of patients with AR every 2 years for 8 years to investigate spirometric abnormalities/BHR	34 of 89 AR patients developed BHR after 8 years Sensitization to mite, birch and parietaria, as well as rhinitis duration are risk factors
Navarro et al. [17]	Epidemiologic prospective; multi centre	942 (with asthma)	Mean age: 35.5; 63% female	Investigate the link between the upper and lower airways	89.5% had AR Correlation between severity of rhinitis and asthma (p < 0001) and inverse correlation with age (p < 0.0001) and severity of asthma (p < 0.05)
Ko et al. [18]	Cross sectional; questionnaire	600 (with asthma)	267 male; 333 female	Evaluation of prevalence of AR in asthma	77% of asthmatics had rhinitis in the past 12 months (of whom 96% were previously diagnosed with AR)
					In patients with asthma and rhinitis, 49% use nasal steroids, resulting in fewer ED visits (13 vs 25%) and fewer hospitalizations for asthma (5 vs 13%)
Valero et al. [19]	Cross-sectional international population study; based on questionnaire	3225; 1 positive skin test	Age range: 10–50; 53% male	Evaluation of the link between AR, asthma and skin test sensitization	Asthma presents in 49% of AR patients
					Asthma severity was associated with length of time from onset and with allergic rhinitis severity
					Patients with asthma have a higher number of allergen sensitizations and higher sensitization intensity than those without asthma (p < 0.01)

### Atopic dermatitis (AD)

#### Extent of co-occurrence

In children, there are clear data concerning the co-occurrence of AD and AR, largely from birth cohorts [20].

In one Taiwanese study [21], AR was the most common concomitant atopic disease associated with AD. The group with AD and AR was shown to be more likely to have serum mite-, cockroach- and feather-specific IgE, whereas the positive rates for wheat, peanut and soybean were higher in those with AD without rhinitis.

In a Croatian study [22], the age at onset was younger in the group of AD patients with concomitant AR, suggesting that AD multi-morbidity (although part of the allergic march) may be irrelevant to the later development of isolated respiratory allergy.

Taken together these observations suggest that sensitization in later onset allergy is not via the skin, but by inhalant allergens acting via the respiratory tract mucosa [2].

#### Effects of co-occurrence

While some data suggest that the greater extent of allergic disease promotes more severe reactions [23, 24], other researchers note an inverse relationship between exacerbations in skin and in the respiratory tract [25].

In patients with asthma, AD and AR, the risks of systemic glucocorticoid bioavailability are highest, since treatment is likely to be directed to three sites: skin, bronchi and nose. Absorption occurs (from least to greatest rate) in the nasal mucosa, followed by the bronchi, then the skin. Alternative means of treatment (such as allergen avoidance, saline douching, antihistamines, anti-leukotrienes, immunotherapy and anti-cytokines) may need to be considered.

### Food allergy

#### Extent of co-occurrence

AR can be associated with primary food allergy; however, it is more frequently associated with secondary food allergy, also known as pollen food syndrome (PFS). Some medical professionals refer to pollen food syndrome as oral allergy syndrome (OAS), although strictly speaking the two are not the same. When the term OAS was first used in 1987 it had no connection with pollen allergy but referred to any allergic symptoms in the mouth that often preceded more serious symptoms. The term pollen food syndrome is preferred when referring to those allergy symptoms to food that are linked to pollen allergy, are limited to the mouth and throat, and are usually mild. [26]. The most typical example is the cross-reactivity in patients with birch pollen AR who develop oral symptoms when eating apples, hazelnut, celery, etc. Typically, when these foods are cooked or processed they can be eaten without causing allergic reactions. Allergic reactions in secondary allergy are usually less severe than in primary food allergy [27]. Common symptoms, which usually come on immediately, include: redness, mild swelling or itching of the lips, tongue, inside of the mouth, soft palate and ears, itching and mild swelling affecting the throat. Occasionally, people might also experience symptoms in the oesophagus (gullet) or stomach, causing abdominal pain, nausea and even vomiting. Sneezing, runny nose, or eye symptoms can also occur.

Those sensitised to both birch and grass pollens are more likely to develop pollen food syndrome [28, 29]. They may also experience symptoms to a wider range of fresh fruits and raw vegetables than those who are sensitised to birch pollen alone.

In one Italian study of pollen sensitive AR subjects aged 4–18 years old, a longer AR duration was significantly associated with moderate-to-severe AR symptoms (p = 0.004), and with co-morbidities such as asthma (p 0.030), PFS was present in 24% (p < 0.001) [30]. In a study of 110 UK adults with spring hay fever, 52 participants (47%) were diagnosed with PFS, which is the commonest form of food allergy in the UK [29].

Interestingly, birth order effects differ for different allergic disorders in a large Japanese study of 14,669 schoolchildren aged 7–15 years [31]. There was no significant difference in the prevalence of BA or AD according to birth order, whereas the prevalence of AR, allergic conjunctivitis and FA decreased significantly as birth order increased. This raises questions about the effects of hygiene on different allergic manifestations.

#### Treatment

PFS is usually a mild type of food allergy that occurs upon contact of the mouth and throat with raw fruits or vegetables containing epitopes also present in a pollen to which the subject is sensitized [32].

PFS is a problem in patients sensitized to various pollen allergens. There was clear association between PFS and polyvalent airborne allergy (69%). Cross-reactivity patterns were typical (for example, tree pollen allergy—intolerance of apples, carrots and potatoes; grass pollen allergy—intolerance of kiwi fruit and tomatoes). Subcutaneous SIT significantly alleviated PFS symptoms associated with ingestion of the responsible fruit and vegetables in patients [33].

The treatment is avoidance of the food(s) causing reaction(s). Adrenalin is only indicated if severe reactions are described in the medical history, or if there are reactions to processed food. For primary food allergy an experimental study of SIT using peanuts was suspended because of adverse reactions [34].

SIT for food allergy is reaching the point where it may soon be used routinely in clinical practice. Sublingual immunotherapy is effective for desensitization with a very favorable adverse event profile. Epicutaneous immunotherapy is also effective, most notably in younger children, with a high rate of local reactions. Oral immunotherapy demonstrates high efficacy, but with a higher risk of gastrointestinal and systemic adverse events. The need for long-term application to sustain desensitization is currently unclear. Immunomodulatory adjuvants may be added to enhance or diminish the immunogenicity of proteins, whereas genetic modifications of food allergens are designed to limit the risk of adverse reactions and address the issues of standardization and supply [35].

The most common pollen-fruit cross-reaction is the birch-apple syndrome [36]. Mauro et al. [36] investigated patients with birch-apple syndrome to evaluate the outcome of subcutaneous immunotherapy (SCIT) and sublingual immunotherapy (SLIT). Two of 8 SCIT-treated patients (25%) and 1 of 7 SLIT-treated patients (14.2%) developed complete tolerance to apple. In the remaining patients, an increase in the provocative dose was found in 3 of the SCIT-treated (37.5%) and 2 of the SLIT-treated patients (28.6%). They concluded that different doses of birch extract may be needed in different patients to improve the associated apple allergy and that a finer diagnostic work-up in selecting patients with birch-apple syndrome who are candidates to respond to birch pollen IT also concerning apple allergy is required [36].

### Eosinophilic oesophagitis (EoE)

#### Description

EoE is currently defined as a “chronic, immune/antigen-mediated esophageal disease characterized clinically by symptoms related to esophageal dysfunction and histologically by eosinophil-predominant inflammation” [37].

EoE is a clinicopathologic disorder diagnosed by clinicians taking into consideration both clinical and pathologic information:

- Symptoms related to esophageal dysfunction;
- Eosinophil-predominant inflammation on esophageal biopsy, which is required for diagnosis, characteristically consisting of a peak value of ≥15 eos per high power field (eos/hpf) [38];
- Response to treatment (dietary elimination; topical corticosteroids) supports, but it is not required, for diagnosis (Strong recommendation, low evidence) [37].

#### Extent of co-occurrence

EoE is commonly associated with other atopic diatheses (e.g. food allergy, asthma, eczema, chronic rhinitis, environmental allergies) [39]. In adults, solid food dysphagia is the most common presenting symptom [40, 41], with food impaction necessitating endoscopic bolus removal occurs in 33–54% of adult EoE patients [42]. Other symptoms in adults include chest pain, heartburn and upper abdominal pain [38, 43].

#### Treatment

- *Acid suppression* Approximately one-third of patients with suspected eosinophilic esophagitis have a good clinical and histologic response to proton pump inhibitors (PPIs alone, suggesting that GERD, or a PPI-responsive form of esophageal eosinophilia, may be responsible [44, 45].
- *Dietary therapy* is an effective treatment for eosinophilic esophagitis in children and adults. Dietary therapy is based upon the observation that patients with eosinophilic esophagitis have high rates of food allergies, and that those allergies may contribute to the development of eosinophilic esophagitis [44].

*Topical corticosteroids* have been proven to be an effective therapy for EoE and are a first-line therapy. Whilst available as multi-dose inhalers or aqueous nebulizer solutions for use in asthma, to treat EoE the medication is swallowed rather than inhaled to coat the esophagus and provide topical medication delivery (Recommendation strong, evidence high) [38].

Oral prednisone (a synthetic corticosteroid drug) may be useful to treat EoE if topical steroids are not effective or in patients who require rapid improvement in symptoms (Recommendation conditional, evidence low) [38].

Patients without symptomatic and histologic improvement after topical steroids might benefit from a longer course of topical steroids, higher doses of topical steroids, systemic steroids, an elimination diet or esophageal dilatation (Recommendation conditional, evidence low) [38].

- *Esophageal dilation* Dilation of esophageal strictures is effective for relieving dysphagia, but has no effect on underlying inflammation [46, 47].
- *Other experimental treatments* Prostaglandin D2 receptor antagonist [48], leukotriene inhibitors (Montelukast) [49], Mepolizumab: humanized monoclonal antibody against interleukin (IL)-5 [50], purine analogues **(**Azathioprine or 6-mercaptopurine) [44, 51].

### Allergic conjunctivitis

#### Extent of co-occurrence

Allergic conjunctivitis is the typical conjunctival reaction in AR. It occurs following exposure to allergens. Ocular symptoms occur in 50–70% of patients with rhinitis, being more common with outdoor than with indoor allergens [52]. Pitt et al. [53] reported that seasonal allergic conjunctivitis (SAC) is associated with significant reductions in both ocular and general quality of life.

#### Effects of co-occurrence

Eye symptoms include itching, watering, redness and difficulty with vision due to these. Ocular symptoms are reduced by nasal air filters [54], suggesting that some eye involvement is secondary to nasal reflexes.

#### Treatment

Some INS reduce eye symptoms as well as nasal ones, the recent molecules appear more consistently effective [55]. The combination of intranasal antihistamine plus INS shows greater efficacy on rhinoconjunctivitis [56]. The topical ocular antihistamines, antazoline, azelastine, and emedastine, provide rapid relief of the symptoms of allergic conjunctivitis [57].

### Rhinosinusitis

#### Extent of co-occurrence

The extent of co-occurrence of rhinosinusitis is disputed and is likely to be different in acute rhinosinusitis (ARS), chronic rhinosinusitis (CRS) and CRS with nasal polyposis.

#### Effects of co-occurrence

When considering the role of allergy in sinus disease, it can be speculated that nasal inflammation induced by IgE-mediated mechanisms favours the development of acute and/or chronic sinus disease [58, 59].

Several mechanisms could explain the link between allergic inflammation and sinus disease. Allergic inflammation of the nasal mucosa may give rise to mucosal congestion leading to impaired mucus drainage at the ostiomeatal complex in predisposed patients. Ostiomeatal pathology is considered to be essential to the generation of sinus-related symptoms [59].

Certainly, AR sufferers experience common colds (which are a form of acute rhinosinusitis) more severely and for longer than those without underlying minimal persistent inflammation [11]. The contribution of AR to chronic rhinosinusitis is less clear (EPOS 2012) [9]. One study has demonstrated that, in children, the degree of atopy (as reflected by the number of aeroallergen sensitivities or the presence of atopic multi-morbidities) is not associated with progression to CRS in the pediatric age group [60].

Symptoms of IgE-mediated allergic inflammation should be asked for during history taking in patients with CRS and specific allergy evaluation should be performed in case of clinical suspicion. With regard to treatment, it is recommended that anti-allergic therapy is added to the treatment of patients with chronic sinus disease and concomitant allergy [61].

#### Chronic rhinosinusitis with nasal polyps (CRSwNP)

Chronic rhinosinusitis with nasal polyps (CRSwNP) is associated with high concentrations of IgE in nasal polyp (NP) tissue. CRSwNP often coexists with asthma and this group is particularly characterized with tissue eosinophilia and high local IgE levels [62]. Therefore, an allergic aetiology of NPs has been presumed, though never firmly demonstrated. Between 0.5 and 4.5% of subjects with AR have NPs, which compares with the normal population [9]. In a retrospective study by Settipane and Chafee [63], the nasal polyps were present in 4.2% of the total population of 4986 subjects. Nasal polyp frequency rate was 6.7% in asthmatic patients and 2.2% in the rhinitis alone group. Of the total 211 cases of nasal polyps, 71% had asthma and 29% had rhinitis alone [63]. Pang et al. [64] reported that food allergen intradermal tests were more positive in nasal polyp patients (81%) compared to controls (11%).

In mucosal tissues, mRNA for the ε-chain of IgE was associated with a significant proportion of B cells [65, 66]. Recent evidence has shown local IgE synthesis, local receptor revision, class switch recombination and B-cell differentiation into IgE-secreting plasma cells in NPs [64]. In NPs, the level of IgE is independent of the atopic status of the patient [65, 67, 68] whereas specific IgE in NPs is only partly related to skin prick test positivity [65, 67, 68]. The local IgE in NPs is the result of two types of IgE production: systemic allergic IgE formation and a local polyclonal IgE formation [65, 66]. Local polyclonal IgE correlates with the presence of *Staphylococcus aureus* enterotoxins (SAE) [9, 65–67]. Finally, Gevaert et al. [69] demonstrated that antagonizing IgE by injections of omalizumab is effective for both allergic and nonallergic CRSwNP. The later finding proves the relevance of local mucosal IgE.

CRSwNP is an IgE mediated disease; however, the role of atopy is less clear.

### Otitis media with effusion (OME)

#### Extent of co-occurrence

In one hospital population of children with chronic OME, over 80% had rhinitis [70]. A population survey in Slough schools (in the UK) also demonstrated an association between OME type symptoms and rhinitis [71].

OME is much rarer in adults and is usually found in association with more severe rhinosinusitis (such as aspirin exacerbated respiratory disease, allergic fungal sinusitis and Churg Strauss syndrome) rather than with rhinitis [9].

#### Effects of co-occurrence

The Eustachian tube exerts a major function in middle ear homeostasis via its role in the ventilation and protection of the middle ear and mucociliary clearance. The Eustachian tube contains an allergic inflammatory infiltrate in AR patients [72]. It is therefore not surprising that allergic inflammation with concomitant mucosal swelling may impair the function of the Eustachian tube [73].

Concomitant occurrence of allergic diseases and primary immune deficiencies was reported by Klemola [74]. Klemola [74] reported that 50% of the children with atopic disease had selective IgA deficiency (sIgAD). In allergic diseases such as asthma, atopic dermatitis, allergic rhinitis, and conjunctivitis, there is predisposition to infections resulting from an immunodeficiency [75]. Immune deficiencies and infections may contribute to the development of OME.

#### Treatment

Because of the pathophysiological associations of AR with OME, treatments focusing on allergic inflammation may be helpful in the management of OME [76]. A meta-analysis of 16 randomized controlled trials demonstrated no significant benefit from antihistamines, decongestants, or combined antihistamines and decongestants versus placebo for treatment of OME [77]. Intranasal corticosteroids did reduce the need for surgery in a double blind study in which autoinflation of the middle ear was also effective, but the combination of the two was less efficacious [78].

Atopic status and nasal disease should be evaluated in recurrent or chronic OME patients who have had no response to antibiotic therapy, and INS could be used as an adjunct to treatment of some OME patients. Further studies are needed to elucidate whether atopic status or rhinitis itself may influence the development of OME and the extent of ear involvement in various forms of rhinosinusitis.

### Adenoid hypertrophy (AH)

The adenoid tissue is a peripheral lymphoid organ located in the nasopharynx, forming part of Waldeyer’s ring. It contributes to the development of immunity against inhaled micro-organisms in early life. The volume of the adenoid increases with age and is maximal at 5–6 years, followed by a gradual decrease in volume by the age of 8–9 years [79]. Symptoms related to AH are nasal obstruction, open mouth breathing and snoring. ‘Adenoid face’ can be caused by AH or by severe obstructive rhinitis [61].

#### Extent of co-occurrence

Children with AR appear to have a greater susceptibility to AH than non-allergic children, with IgE-mediated inflammation of the nasal mucosa likely playing a role in both conditions [80].

Adenoid hypertrophy in adults is rare and may indicate underlying malignancy or infection, such as human immune deficiency virus (HIV), rather than allergy.

#### Treatment

Treatment of AR with intranasal corticosteroids has been shown to improve various parameters associated with adenoid hypertrophy [81].

### Olfactory dysfunction

#### Extent of co-occurrence

In a recent study of 51 subjects with AR, half had hyposmia [82]. Half of AR subjects with a normal CT scored in the 30th percentile in an olfactory test. The degree of olfactory dysfunction does not seem to be related to the degree of nasal obstruction/nasal resistance [82], although with perennial (persistent) AR, the olfactory dysfunction seems to be more stable and more persistent throughout the year [83].

Reduced olfaction is more common in chronic rhinosinusitis, especially when NPs are present [9].

#### Effects of co-occurrence

AR may have a negative impact on olfactory function and is considered to be a sinonasal-related origin of chemosensory dysfunction [84]. This is probably secondary to a local inflammation in the nasal fossa and around the olfactory neuroepithelium in the olfactory cleft rather than a nasal obstruction impairing the odours to reach the olfactory cleft [82].

Olfactory function is a global perception of orthonasal and retronasal stimuli and of a stimulation of intranasal trigeminal nerve endings. The trigeminal function is enhanced in patients with AR and a link exists between chemosensory trigeminal function and neuroinflammation [85]. Antidromic activation of the trigeminal nerve after allergen and/or chemosensory stimuli leads to the liberation of neuropeptides and classical trigeminal related symptoms such as sneezing and itching.

#### Treatment

Systemic corticosteroid is the first-line-treatment for olfactory dysfunction of sinonasal origin but use must be short term because of side-effects. Topical corticosteroid does not reach the same efficacy but has a good safety profile [86]. Nasal corticosteroids have a positive impact on AR-related olfactory dysfunction [87]. However, most studies have used a visual analogue scale to evaluate olfactory function, which is known to be a poor tool in comparison with other test modalities such as threshold, discrimination or identification tasks in olfactory testing.

Antihistamines also have a positive effect on the olfactory function even if prescribed for a short period of time [88]. Olfactory dysfunction has marked effects on quality of life when severe and needs to be taken into account in the evaluation of comorbidities related to the presence of AR.

### Laryngitis, cough and vocal problems

#### Effects of co-occurrence

The passage of mediators, cytokines and secretions backwards from the nose should not reach the larynx, unless epiglottic function is disturbed. However, many patients with AR do experience throat symptoms, including irritation, the sensation of difficult to shift mucus and cough [89]. In a recent study on AR and laryngeal symptoms, involving six controls and six adult singers, nasal provocation with pollen extracts caused a rapid induction of laryngeal irritation and globus sensation. However, no objective changes occurred then, nor during the pollen season [90].

#### Treatment

Edema of the laryngeal mucosa, laryngeal erythema and candidiasis may all be found in a minority of patients treated with inhaled glucocorticosteroids [91], but are not reported after the prolonged use of a nasal steroid spray.

### Gastro esophageal reflux (GER)

Gastro esophageal reflux (GER) may masquerade as CRS [92]. Associations have been reported between GER and a variety of upper and lower respiratory tract conditions but not with AR [73].

### Obstructive sleep apnea (OSA) and sleep impairment

The effect of AR on sleep can impair quality of life [93]. Patients with AR have more difficulties in falling asleep, take more sleeping drugs, suffer from nocturnal awakenings, and feel that they do not get sufficient sleep when compared to healthy controls [94].

#### Treatment

One study [93] has demonstrated an improvement in OSA in a short-term trial of intranasal fluticasone propionate. The mixed/obstructive apnea-hypopnea index decreased by 4.9 ± 1.0 events per hour in the fluticasone propionate group and increased by 2.2 ± 3.3 events per hour in the placebo group [94]. Craig et al. studied a group of 20 adults with perennial rhinitis and sleep complaints, examining the benefit of twice-daily nasal flunisolide using a double-blind, placebo-controlled, crossover study. They found that nasal congestion and subjective sleep improved significantly in the topical corticosteroid-treated subjects. They concluded that the fatigue in perennial allergies may be a result of nasal congestion and associated sleep fragmentation. Decreasing nasal congestion with nasal steroids may improve sleep, daytime fatigue, and the quality of life of patients with AR [95].

### Fatigue and learning impairment

#### Extent of co-occurrence

Patients with AR frequently complain of disordered sleep, daytime somnolence and inability to concentrate. Recent studies document daytime somnolence in children with AR. Craig et al. [95] reported an association between daytime somnolence and nasal congestion in a group of patients with AR. If nasal symptoms such as itching, sneezing, rhinorrhea, and congestion are not well controlled during the day, they may contribute to learning problems during school hours. If these symptoms are not well controlled during the night, they may contribute to nocturnal sleep loss, secondary daytime fatigue and learning impairment [96].

#### Effects of co-occurrence

Baraniuk et al. [97], in a study of chronic fatigue syndrome, failed to document an increase in AR in these patients. However, patients with AR (unlike the group with rheumatologic disease) had significantly increased symptoms of fatigue intermediate between levels of fatigue seen in normal subjects and patients with chronic fatigue syndrome.

AR can reduce driving performance [98]. It can also impair examination performance in adolescents [99].

#### Treatment

In a study involving major examinations, sedating antihistamines increased the likelihood of dropping an examination grade [99]. The medications used to treat allergic rhinitis may cause central nervous system adverse effects and contribute to learning impairment. The newer relatively nonsedating medications such as loratadine, cetirizine, and fexofenadine have less potential to impair central nervous system function and learning than their predecessors [96].

### Turbinate hypertrophy

#### Extent of co-occurrence

In AR patients, nasal obstruction is a bothersome symptom which is most commonly due to inferior turbinate hypertrophy. The inferior turbinate is the initial deposit point for allergens and undergoes dynamic changes through the allergic cascade, which results in nasal obstruction. Targeting the inferior turbinate to augment the nasal airway is the mainstay of surgical treatment in AR [100].

#### Effects of co-occurence

The turbinates are tiny shelf-like bony structures that project into the nasal passageways. They help warm, humidify, and clean the air that passes over them. If turbinate hypertrophy develops, it causes persistent nasal congestion and, sometimes, pressure and headache in the middle of the face and forehead. This condition may require surgery [101].

#### Treatment

Turbinate hypertrophy which is multimorbidity of the AR is primarily treated by allergen avoidance and medical treatment, but when these measures fail to control symptoms then surgery to the inferior turbinates of nose can be performed [102]. Outfracture, submucous resection, laser vaporization, radiofrequency ablation, and coblation, cryosurgery, submucous electrocautery, and microdebriber turbinoplasty are treatment options in turbinate hypertrophy related to AR [103].

## Diagnosis

Diagnosis of multi-morbidities of AR are shown in Table 2 [3].

**Table 2:** Diagnosis of multi-morbidities associated with allergic rhinitis (AR)

Multi-morbidities of AR	Definitive medical history, symptoms and signs
Asthma	Ask about any history of cough, wheeze, shortness of breath, exercise-induced bronchospasm
	Examine the chest for wheeze, hyperexpansion
	Assess peak expiratory flows and spirometry in older children preferably with reversibility testing with beta-2 agonists
	If in doubt, undertake an exercise, mannitol or methacholine challenge test or measure exhaled nitric oxide (FENO)
Conjunctivitis	Ask about a history of red, itchy, watery eyes, eye rubbing
	Examine eyes
Rhinosinusitis	Ask about a history of nasal obstruction or discharge (purulent) with or without hyposmia, headache, facial pain or cough
	Undertake nasendoscopy in older children
	CT scan/sinus X-rays not recommended unless there are complications or failed therapy, unilateral symptoms or severe disease unresponsive to medical therapy
Otitis media with effusion (OME)/impaired hearing	Ask questions related to immune deficiency and/or recurrent infections
	Ask about any speech and language delay, increasing volume of TV, shouting, poor concentration, failing performance at school, frustration, irritability
	Examine the ears using a pneumatic otoscope if possible, and Weber and Rinne tests
	Use tympanoscopy for evaluation of tympanic membrane and middle ear
	Undertake tympanometry
	Use a whisper test to screen otitis media with effusion and hearing loss
	Use audiometry in older children—pure tones, speech
Obstructive sleep apnea and sleep problems	Enquire about any history of disturbed sleep, snoring, apnoea, tiredness, irritability
	Assess nasal airway using spatula misting, nasal inspiratory peak flow, visual examination of nostrils and nasendoscopy in older children to view nasal airway and adenoids
	Consider sleep study
Atopic dermatitis	Ask about skin symptoms of itching, redness, rash
Food allergy	Ask about symptoms related to food intake
	Ask for oral allergy syndrome (OAS): Allergic reaction that occurs upon contact of the mouth and throat with raw fruits or vegetables which may be tolerated when cooked
Eosinophilic oesophagitis	Ask for symptoms related to esophageal dysfunction as solid food dysphagia, chest pain, heartburn and upper abdominal pain
	Assess esophageal biopsies
Adenoid hypertrophy	Ask about nasal obstruction, open mouth breathing and snoring
	Examine the face
	Perform posterior rhinoscopy; nasal and nasopharyngeal rigid/flexible endoscopy
Olfactory dysfunction	Ask for olfactory dysfunction, hyposmia, anosmia
	Evaluate nasal airway and smell function tests
Laryngitis, cough and vocal problems	Ask for symptoms including irritation in the throat, the sensation of difficult to shift mucus and cough
	Examine throat and larynx, see vocal cords and arytenoids
Gastro esophageal reflux	Ask for symptoms of indigestion, regurgitation, cough
	Examine throat and larynx
Fatigue and learning impairment	Ask about fatigue and learning impairment, school success
	Ask about sleep quality, nasal obstruction and nasal discharge
Turbinate hypertrophy	Ask about nasal obstruction
	Perform anterior rhinoscopy and nasal endoscopy, acoustic rhinometry pre and post decongestant shows whether mucosal lining or bony structure is responsible

A physical examination of all organ systems potentially affected by allergies, with emphasis on the upper respiratory tract, should be performed in patients with a history of rhinitis.

## Treatment

**Fig. 1:**
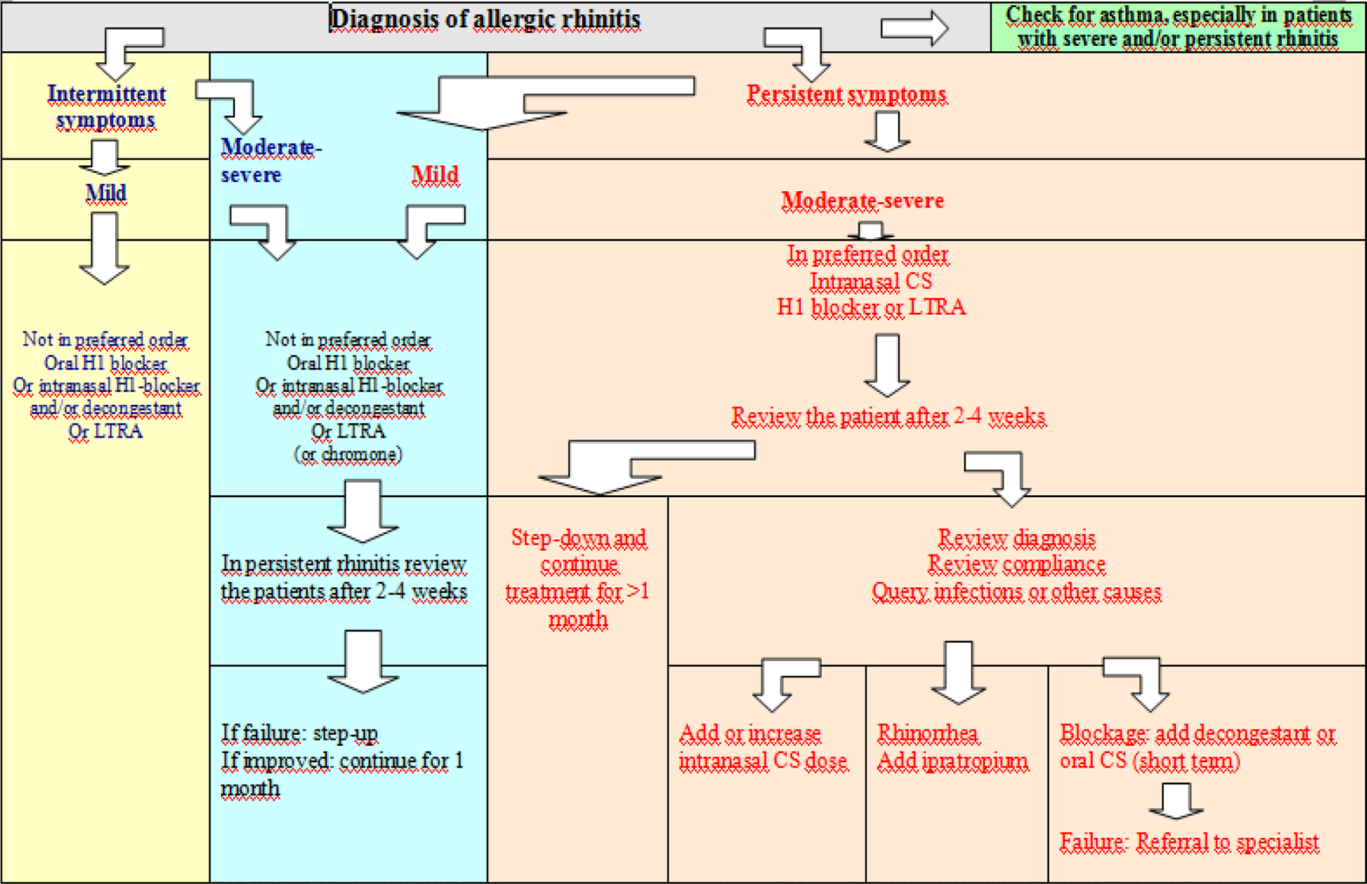
Treatment for AR (taken from ARIA 2012) [104]. In addition to the pathways presented in the figure allergen and irritant avoidance may be appropriate; for conjunctivitis, add an oral H1-blocker, intraocular H1-blocker or intraocular cromone (or saline); consider specific immunotherapy when pharmacotherapy fails or is unacceptable to the patient

During the treatment of AR, multi-morbidities (co-morbidities) of AR should be considered. Treatment of AR according to guidelines may cause decreasing the nasal symptoms; and may cause improvement of the co-morbid problems; see Fig. 1 [104].

## Conclusion

This EAACI Task Force Report on AR Multi-morbidities has defined and classified the multi-morbidities associated with AR, together with providing recommendations for diagnosis and treatment. The information provided here should help improve allergists’ knowledge and skills, ultimately improving the overall quality of patient care, as well as helping to raise the profile of this important area of work.
